# When it is time to hang up the keys: the driving and dementia toolkit – for persons with dementia (PWD) and caregivers – a practical resource

**DOI:** 10.1186/1471-2318-13-117

**Published:** 2013-11-01

**Authors:** Anna Byszewski, Faranek Aminzadeh, Kelly Robinson, Frank Molnar, William Dalziel, Malcolm Man Son Hing, Lynn Hunt, Shawn Marshall

**Affiliations:** 1 The Ottawa Hospital, Regional Geriaric Program of Eastern Ontario, 1053 Carling Avenue, Ottawa, ON K1Y 4E9, Canada; 2 Ottawa Hospital Research Institute, 725 Parkdale Ave, Ottawa, ON K1Y 4E9, Canada; 3 Department of Medicine, Faculty of Medicine, Division of Geriatric Medicine, University of Ottawa, 451 Smyth Road, Ottawa, ON K1H 8 M5, Canada; 4 Rideau Community Health Services, 354 Read Street, P.O. Box 550, Merrickville, ON K0G 1 N0, Canada; 5 The Rehabilitation Centre, The Ottawa Hospital, 505 Smyth Road, Ottawa, ON K1H 8 M2, Canada; 6 The Ottawa Hospital, Geriatric Assessment Unit, 1053 Carling Avenue, Box 678, Ottawa, ON K1Y 4E9, Canada

## Abstract

**Background:**

The aim of this project was to develop a toolkit to assist persons with dementia (PWD) and their caregivers, in planning for retirement from driving. The information gathered was used to develop a tool that can assist reflection about, and make sound decisions in this challenging area of the dementia journey. The purpose is to keep safe drivers on the road and to prepare those who are moving towards being at risk of being involved in crashes, to eventually stop driving when they are unsafe.

The toolkit was prepared to address the concerns of both the PWD as well as the caregivers. Strategies and solutions are presented for both the PWD and the caregivers. A grief insert was also developed that can assist caregivers in supporting the PWD in the grief process that can accompany losing one’s driving privileges.

## Scope of the problem

Demographics suggest as the populations age, there will be more older drivers on the road, with the number of licensed drivers expected to double [1]. Motor vehicle accident rates are reported to increase with the aging population; Lyman [2] predicted that between 1999 and 2030, there would be a 155 percent increase in fatal crash rates, and a 178 percent increase in all police reported crash involvements in drivers older than 65 years of age. Also as multiple co-morbidities accrue with age, it is well recognized that these can impact safety driving. [3] In particular, cognitive deficits such as dementia, can negatively affect the ability to drive safely [4]. Marshall and Man Son Hing [5] found that most older drivers are proactive and will self regulate and restrict driving or cease driving all together, especially as they develop multiple medical conditions. Examples of self-regulation include an awareness of age and health-related changes in driving ability, reporting monitoring own driving safety, adopting strategies to decrease risk and expressing a willingness to consider changing their driving behavior to improve safety [6-9]. However dementia can negatively affect the ability to self-reflect and to be insightful as to one’s safety driving and appropriately self-regulate, therefore some PWD continue to drive despite their disease progressing beyond the point where driving is still safe [10]. Dementia causes not only loss of memory but also affects other areas of cognition that are important for driving safety. These include ability to shift attention, problem solving skills, orientation, judgement and speed of reaction [11]. Often the PWD may not be aware of these difficulties [12] and it is those around them that notice these changes, including friends and caregivers [13]. Despite older adults self regulating by driving shorter distances and avoiding traffic, most accidents still occur close to home, such as on trips to the grocery store, in mall parking lots or on the way to church on low-speed stretches, and with left turns and intersections [14].

### PWD and caregiver opinions

Most PWD do not plan to retire from driving and express the goal of driving for as long as possible [7]. It is perceived as one of the most difficult decisions they ever have to make, with an accompanying sense of loss of meaning of life [15]. For many PWD there is an intense fear of being discriminated against: the “older driver stigma”. There are concerns that admitting to a decline in ability and self-regulation, may become **“**red flags” and penalize them, resulting in loss of their driver’s license. Many express skepticism that they may not receive the “right” feedback and fear the abrupt and imposed loss of driving privilege.

In many instances, PWD have difficulty accepting the decision, and perceive the assessment results more as nebulous, often questioning the evaluation process [15]. As a result of losing their license, the PWD may have to rely on rides from family and friends, although they dislike this position of dependence [16].

### Possible solutions for PWD and their caregivers - assessment stage

There is emerging evidence that advanced planning is critical for a gradual reduction in driving and eventual cessation. Previous research [17] by the authors has indicated that PWD prefer a warning and a gradual discussion centered around driving, and usually preferred that this come from the physician. PWD seek information and need reaffirmation why they are unsafe to drive. When cognitive difficulties emerge, if possible, it is preferable that health professionals discuss driving safety long before it becomes a safety issue, and thus avoiding a sudden recommendation and confrontation. It is important to assess where older individuals are within this process, and address the individual’s needs accordingly. A proactive and enabling approach with timely discussion and consultation with all involved, allows for empowering the decision making process for the PWD experiencing cognitive loss and their caregivers. Mobilizing family and community support and adequate follow up are strategies that can ensure a successful transition. Work by Dobbs et al. [18] has shown that driving cessation groups designed to assist in the driving retirement process can mitigate the negative consequences of losing one’s driving privileges. Framing driving as a health issue and not a “taboo” topic can assist in shifting the focus from age to risk factors. Explaining the reasons for the recommendations, including results of tests and their impact on driving ability can further understanding and acceptance. Focusing on physical problems (e.g. vision loss or medications), may make the driving cessation more acceptable to the PWD [17]. It is important to listen attentively, empathize and problem-solve to minimize the impact on quality of life. One strategy can be to shift the tone from confrontation to collaboration, by broadening the discussion from determining the fitness to drive to driving retirement, education and support. Appropriate referral to counseling and support groups and arranging follow up to address possible grief and depression can mitigate the discussion. Work by Aminzadeh et al. [16] demonstrated the importance of acknowledging losses and empathizing, as the PWD can exhibit a range of powerful reactions, ranging from shock and disbelief to anger, sadness and helplessness. Other resources include workbooks, self assessment tools (in early stages of dementia), presentations at service clubs and on-line resources that are designed to increase awareness of potential problems, and facilitate communication [19-22].

### Alternative transportation plan and adjustment - after driving cessation

Often the PWD and their caregivers have to adjust without sufficient support and information during the transition to non-driver status and are unaware of driving alternatives [23]. The PWD can benefit from a plan for alternative transportation: the plan should be concrete, individualized and situation specific. It may help to provide information verbally and in writing to enhance understanding. The ingredients for successful transition optimally would include: planning ahead for gradual reduction and eventual cessation of driving, voluntary/involved cessation decision, a decision that is appropriately timed, and access to acceptable alternative mobility options. This can include volunteer drivers, mobility assisted transportation or taxis and family/friend transportation. Research is emerging that support groups can help PWD work through their emotions and ameliorate some of the negative emotional impact (e.g., anger, surprise, depressive symptoms, etc.) using various measures. [18] Changes in driving identity may require negotiation and acceptance over time, with family input and sensitivity [7].

### Aim of this project

The aim of this project was to develop a much needed evidence-based and comprehensive toolkit to assist PWD/caregivers in planning for retirement from driving. The information gathered was used to develop a tool that can assist reflection about, and make sound decisions in this challenging area of the dementia journey. The purpose is to keep safe drivers on the road and to prepare those who are moving towards being at risk of being involved in crashes, to eventually stop driving when they are unsafe. The toolkit was prepared to address the concerns of both the PWD as well as the caregivers. Strategies and solutions are presented for both the PWD and the caregivers. A grief insert was also developed that can assist caregivers in supporting the PWD in the grief process that can accompany losing one’s driving privileges. This is a companion toolkit to the “Driving and Dementia Toolkit for Health Professionals”, a recognized resource in the care of persons with dementia, now in its third edition available in print and on-line [15].

In the development phase the materials for this resource toolkit were developed by an interdisciplinary team of professionals which included representatives from nursing, social work, occupational therapy, geriatric medicine and rehabilitation medicine.

Background materials were developed based on a comprehensive review of the international published and gray literature (informally published literature, that may be difficult to trace via conventional channels, such as working papers and government agency documents) and the authors’ collaborative research [17]. Feedback was sought from discussions held on the topic of driving and dementia, during regular Ottawa and Renfrew County Alzheimer Society chapter support meetings. The toolkit purpose was explained at the beginning of the session, and with the participants’ approval, the toolkit was reviewed and then modified, based on consultation with these partners. As no research was carried on human subjects, research ethics board approval was not sought. No direct quotations or identifiable information was used in production of the toolkit. In addition, resources already developed were gathered for use and were integrated into this project.

### Toolkit development

Review of resources available identified a variety of resources for seniors in general, but demonstrated a significant gap in tools specific to PWD and their caregivers. The AMA Physician Guide to Assessing and Counseling Older Drivers, 2nd edition 2010 [22] and At The Crossroads, Hartford Foundation 2010 are two recent tools that have been developed in the United States [24]. In Canada, The Canadian Medical Association Driver's Guide: Determining Fitness to Operate Motor Vehicles [25] addresses multiple medical issues but is geared towards health professionals, and there is currently no comprehensive tool for PWD and caregivers that addresses driving issues specific to the Canadian context. The review by the Alzheimer Society partners enriched the knowledge base for ensuring a tool that was geared to this specific population. Insights gained in these discussions included the challenges that are faced during this important transition and offered suggestions regarding how professional providers could have been more sensitive and responsive to their needs. Some of the issues raised by these partners consulted included the need to obtain feedback about testing results and for health professionals to be knowledgeable in dealing with the impact of the emotional response. Suggestions also included more discussion by health professionals of alternative transportation strategies and resources.

### Toolkit contents

The toolkit was developed as a paper based booklet, and it is also available on-line (http://www.rgpeo.com/media/30422/d%20%20d%20toolkit%20pt%20crgvr%20eng%20with%20hyperlinks.pdf) and it has been translated into French. It has been proof read for language level appropriateness. It is divided into 5 general sections.

### a. Section 1 - general information

The first section includes general information, including some background information on dementia and driving, warning signs, what to watch for, and explanation of physician responsibilities. It also includes an algorithm (see Additional file 1), which is a “road map” of the assessment process. This section also includes some frequently asked questions on the topic such as the examples provided below:

Q1. Why does dementia make one an unsafe driver?

Answer: Dementia causes not only loss of memory but also affects other areas of our thinking skills that are important when we drive. These include ability to shift attention, problem solving skills, orientation, judgment and speed of reaction. Often the person with dementia may not be aware of these difficulties and it is those around them that notice these changes.

Q2. But I only drive short distances, so why worry?

Answer: Most accidents occur close to home, such as on trips to the grocery store, in mall parking lots or on the way to the church.

### b. Section 2- assessment procedures: How does It All work?

The second section informs PWD/family about the different types of assessments that can be performed, both off road and on the road. These include the physician’s exam in the office, or occupational therapy or neuropsychologist paper based evaluations. Other information includes discussion of simulator testing or specialized comprehensive on the road evaluations that involve the occupational therapist and a driving instructor, and the cost involved.

### c. Section 3- after the assessment: next steps

The third section is linked to the road map algorithm (Additional file 1), and it details the steps after the driving assessment. It is further subdivided into three color coded subsections.

The “green” section speaks to the case if the PWD is found safe to drive, highlighting the need to prepare for eventual driving cessation; interim compensatory strategies and preparing for the time when one must cease driving. The health professionals and caregiver’s role for monitoring and periodic follow up is highlighted.

The “yellow” section addresses those who may have uncertain risk, and may need more evaluation, including more in-depth, specialized assessment and close follow up.

The “red” section details the professional’s responsibility to communicate in a clear and compassionate manner the recommendation to stop driving and suggestions are given for developing an alternative transportation plan. This section emphasizes the possible range of emotional reactions of the PWD, and the safety issues that the caregivers may need to address.

### d. Section 4 - useful resources

The fourth section provides samples of resources which can assist the PWD and the caregiver. It includes the Advance Directive for Driving Cessation, developed by the Hartford Foundation (used with permission for the purpose of this toolkit). It is an example of a letter that can be written in advance to plan for driving retirement with a copy shared with the caregiver(s) and the health professional. It also includes a sample letter (see Additional file 2) that can be given to the PWD, signed by their doctor, explaining why they can’t drive, to serve as a eminder of the discussion. A copy can be provided to the caregiver as a reminder of the discussion, in case the PWD forgets about the directive from the physician. Several on line publication resources are listed as well in this section of organizations who have developed materials geared towards this challenging area. Examples include: The Regional Geriatric Program of Eastern Ontario [26] CanDRIVE [27] and The Alzheimer’s Association [28].

### e. Section 5 - removable pocket section

A removable folder section is available for local resources, such as volunteer driver lists, Para Transpo forms, Alzheimer Society information and local driving assessment sites. A two page grief insert is included, developed as part of this project, which can assist caregivers in supporting the PWD, when driving privilege is lost. This information can be tailored to the local resources available in various regions.

## Discussion

With the aging demographic worldwide, there is an emerging need to consider and plan for eventual *retirement* from driving. This is an important late life transition with far reaching implications for the quality of life of PWD and their caregivers. From the available literature and our own review, it appears that PWD and caregivers may not receive sufficient information, education and support from professional providers to make the right decision, to feel comfortable with their decision and to adjust to the change.

This toolkit can be a helpful resource to PWD and caregivers during this difficult process. It can also facilitate and support the work of professional providers who assist them in this journey. The information contained within this toolkit can increase awareness of potential problems in domains of health, ability, attitudes, and behaviors. It highlights the need for improved communication that can facilitate discussions of issues related to driving cessation between the older person, family and professional providers. This material can also empower PWD and caregivers with a greater degree of self-monitoring and self-regulation of driving behavior.

This material was developed in partnership with the Alzheimer Society in Canada, who have long supported our driving safety initiatives. Similar organizations, including the Alzheimer Association in the United States and beyond North America, worldwide organizations can use and adapt this material.

### Limitations

The toolkit content may be less applicable for reading by those PWD who have more advanced disease, being more relevant in this situation as a resource to the caregiver(s).

## Conclusions

Clearly driving cessation decisions have health, quality of life and safety implications for the older drivers, their family, and the public. This is an example of an innovative tool that can provide better preparedness for this life transition. This toolkit is designed to help persons in the early stages of dementia and their caregivers (family, friends and other support persons). It may also be useful to health professionals as they introduce the topic of driving cessation to their patients with cognitive loss and their caregivers.

## Competing interests

The authors declare that they have no competing interests.

## Authors’ contributions

All authors (AB, FA, KR, FM, WD, MMSH, LH, SM) were involved in the design of the study and toolkit development, as well as manuscript preparation and review. All authors read and approved the final manuscript.

## Pre-publication history

The pre-publication history for this paper can be accessed here:

http://www.biomedcentral.com/1471-2318/13/117/prepub

## Supplementary Material

Additional file 1 Algorithm - Road Map. Click here for file

Additional file 2 Sample Letter to PWD. Click here for file
