# Supplementary material for: When it is time to hang up the keys: the driving and dementia toolkit – for persons with dementia (PWD) and caregivers – a practical resource
Source: BMC Geriatr. 2013 Nov 1;13:117. doi: 10.1186/1471-2318-13-117 (PMC4228426; doi:10.1186/1471-2318-13-117)
Supplement: Additional file 1 — Algorithm - Road Map. [file 1471-2318-13-117-S1.doc]

**Additional file 1: ROAD MAP**

**Possible Steps for Assessment of a Driver with Dementia**

The doctor will confirm the diagnosis of dementia and address any conditions that could be aggravating the situation

Driving risk is clear

The doctor will ask the person to stop driving (may provide a written record and will document in medical record)

Provincial Registrar will be notified and license

will be revoked

Driving risk is uncertain

Referral to a local multidisciplinary dementia assess-ment site-could include occupational therapy or neuropsychology evaluation

Appears

Safe

Follow Up with physician

(every 6 –

12 months)

Referral to a health professional led comprehensive driving evaluation-on/off road

**IF** there are additional health and safety concerns which require further assessment and treatment/patient truly cannot afford on-road test).

**IF** driving safety is the only issue to assess.

If still

unsure re: fitness

to drive
