# Supplementary material for: When it is time to hang up the keys: the driving and dementia toolkit – for persons with dementia (PWD) and caregivers – a practical resource
Source: BMC Geriatr. 2013 Nov 1;13:117. doi: 10.1186/1471-2318-13-117 (PMC4228426; doi:10.1186/1471-2318-13-117)
Supplement: Additional file 2 — Sample Letter to PWD. [file 1471-2318-13-117-S2.doc]

**Additional file 2**

**Sample Doctor’s Written Statement to the Patient**

(*This is an example of a letter the doctor can provide for the person with dementia to remind the person of the need to stop driving. A copy can be given to the family caregiver.)*

Date:

Name:

Address:

Dear Mr. (Mrs.) ___________:

I realize that this is a difficult recommendation for you, but based on the results of tests performed, I am recommending you do not drive.

You have undergone assessment for memory/cognitive problems. It has been found by comprehensive assessment that you have ________________________ dementia. The severity is _________________.

  Even with mild dementia, your risk of a car accident in the next year is eight times that of other people your age. Even with mild dementia, the risk of a serious car crash is 50% within two years of diagnosis.

Additional factors in your health assessment that raise concerns about driving safety include:_______________________________________________________________________

______________________________________________________________________________

______________________________________________________________________________

As your doctor, I have a legal responsibility to report potentially unsafe drivers to the Provincial Registrar. I have no choice in this matter. Even with a previous safe driving record, your risk of a car crash is too great for you to continue driving. Your safety and the safety of others are too important.

M.D.

Date

Copies given to: _________________________________
